# FUS-NLS/Transportin 1 Complex Structure Provides Insights into the Nuclear Targeting Mechanism of FUS and the Implications in ALS

**DOI:** 10.1371/journal.pone.0047056

**Published:** 2012-10-08

**Authors:** Chunyan Niu, Jiayu Zhang, Feng Gao, Liuqing Yang, Minze Jia, Haining Zhu, Weimin Gong

**Affiliations:** 1 Laboratory of Non-coding RNA, Institute of Biophysics, Chinese Academy of Sciences, Beijing, China; 2 Department of Molecular and Cellular Biochemistry & Center for Structural Biology, College of Medicine, University of Kentucky, Lexington, Kentucky, United States of America; University of Florida, United States of America

## Abstract

The C-terminal nuclear localization sequence of FUsed in Sarcoma (FUS-NLS) is critical for its nuclear import mediated by transportin (Trn1). Familial amyotrophic lateral sclerosis (ALS) related mutations are clustered in FUS-NLS. We report here the structural, biochemical and cell biological characterization of the FUS-NLS and its clinical implications. The crystal structure of the FUS-NLS/Trn1 complex shows extensive contacts between the two proteins and a unique α-helical structure in the FUS-NLS. The binding affinity between Trn1 and FUS-NLS (wide-type and 12 ALS-associated mutants) was determined. As compared to the wide-type FUS-NLS (K_D_ = 1.7 nM), each ALS-associated mutation caused a decreased affinity and the range of this reduction varied widely from 1.4-fold over 700-fold. The affinity of the mutants correlated with the extent of impaired nuclear localization, and more importantly, with the duration of disease progression in ALS patients. This study provides a comprehensive understanding of the nuclear targeting mechanism of FUS and illustrates the significance of FUS-NLS in ALS.

## Introduction

Fused in sarcoma/translocated in liposarcoma (FUS/TLS) is a DNA/RNA binding protein that is involved in many processes of RNA metabolism including gene transcription regulation, RNA splicing and transport, and translation [1]–[4]. Wild-type FUS predominantly resides in the nucleus and shuttles between the nucleus and cytoplasm [5], [6]. In addition to its role in oncogenesis, mutations in FUS have been recently reported to cause a familial form of amyotrophic lateral sclerosis (ALS) [7], [8]. Abnormal accumulation of FUS in the cytoplasm and formation of pathological inclusions are a prominent feature observed in both familial and sporadic ALS [7]–[9].

**Figure 1:**
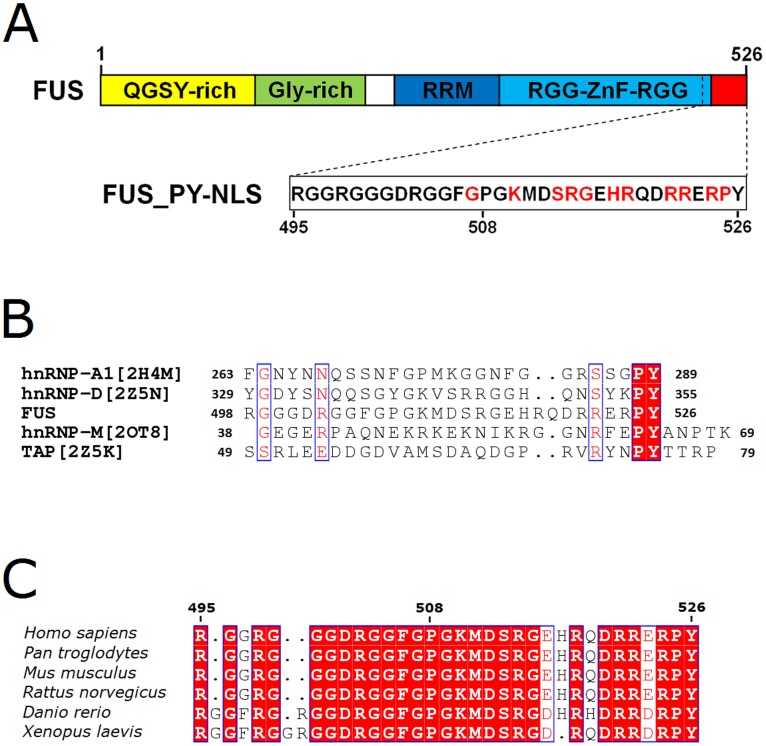
Sequence analysis of FUS-NLS. (A). Domain structure of FUS with the C-terminal NLS. The ALS mutations are clustered in the NLS and the mutations studied here are shown in red. (B) Amino acid sequence alignments of FUS-NLS with other PY NLS’s from hnRNP A1, hnRNP D, hnRNP M and TAP. (C). Sequence alignment of FUS-NLS from different organisms.

Several laboratories, including ours, have identified the FUS C-terminal nuclear localization sequence (NLS) and noted that ALS-associated mutations in FUS are clustered within the NLS (Figure 1A) [10]–[12]. The ALS mutations in the FUS-NLS caused cytoplasmic mis-localization of FUS and induced the formation of FUS-positive cytoplasmic inclusions. Moreover, nuclear import of FUS is dependent on the nuclear import protein transportin 1 (Trn1) [11]. It is thus postulated that the point mutations in the FUS-NLS would block the recognition of FUS by Trn1.

A non-classical PY NLS has been found in other proteins. Several structures of Trn1 in complex with the PY NLS from proteins such as hnRNP A1-NLS (PDB code: 2H4M [13]), hnRNP M-NLS (PDB code: 2OT8 [14]), hnRNP D-NLS and TAP-NLS (PDB codes: 2Z5N and 2Z5K [15]) have been determined. Within Trn1, site A (HEAT repeats 8–13) and site B (HEAT repeats 14–18) are responsible for binding the PY NLS [15]. The previous studies suggest that PY NLS is structurally disordered, overall positively charged, and has a central hydrophobic or basic motif followed by a C-terminal R/H/KX_(2–5)_PY consensus sequence. The last two residues Pro and Tyr are found to be critical for the Trn1 recognition [13]. Other than the Pro and Tyr residues, the FUS-NLS shows obvious differences in amino acid sequences from other known PY NLS’s (Figure 1B). The FUS-NLS only shares 21%, 28%, 18%, and 12% sequence identify with hnRNP-A1-NLS, hnRNP-D-NLS, hnRNP-M-NLS, and TAP-NLS, respectively. Interestingly, the NLS sequence of FUS is highly conserved among different organisms (Figure 1C). Thus, we speculate that the FUS-NLS recognition by Trn1 will possess unique characteristics at the molecular level.

Given the critical significance of the FUS-NLS in regulating its subcellular localization and in ALS pathology, we determined the 3.0-Å crystal structure of the human Trn1/FUS-NLS complex. Our results reveal a well folded FUS-NLS that maintains extensive hydrophobic and electrostatic interactions with Trn1, distinctly different from other PY NLS’s. We also performed surface plasmon resonance (SPR) to measure the binding affinity between Trn1 and wild-type (WT) FUS-NLS or the ALS-associated FUS mutants. When compared with WT FUS-NLS, each of the ALS-associated mutations causes a reduction in the affinity and the range of this reduction varies from 1.4-fold to 714-fold. Moreover, the extent of impaired nuclear localization of the ALS mutants correlates well with the fold reduction in affinity. The results from this comprehensive characterization of FUS-NLS as well as the ALS mutations provide critical insights into the nuclear targeting mechanism of FUS in the context of ALS.

## Materials and Methods

### Protein Expression and Purification

The full-length human transportin 1 (Trn1, residues 1–890, a generous gift from Dr. Yuh Min Chook) was subcloned into the pGEX-4T-3 vector containing a TEV protease-cleavage site and expressed in *E. coli* BL21 (DE3) (Novagen, Madison, WI). Protein purification followed the published protocol [16]. Briefly, cells were harvested by centrifugation, resuspended in lysis buffer (50 mM Tris-HCl, pH 7.5, 100 mM NaCl, 20% glycerol, 2 mM EDTA, 2 mM DTT) and disrupted in a French Pressure Cell. After centrifugation at 38,900 g for 30 min, the target protein was purified by glutathione Sepharose 4 Fast Flow (GE Healthcare, Uppsala, Sweden) and eluted with the lysis buffer plus 20 mM glutathione. After the removal of the GST-tag by TEV protease digestion, Trn1 was further purified by two steps of column chromatography with HiTrap Q FF 5-ml and Superdex 200 HR 10/30 columns (GE Healthcare).

The cDNA encoding the nuclear localization sequence of human FUS (FUS-NLS, residues 495–526) was amplified by PCR using the GFP-FUS plasmid template we previously published [10] and subcloned into pGEX-6P-2 to include an N-terminal GST tag. The GST-FUS-NLS fusion protein was expressed in *E. coli* Rosetta (DE3) (Novagen) and purified similarly as for Trn1. The difference is (i) after glutathione-affinity column, PreScission protease (GE Healthcare) was applied to cleave the fusion FUS-NLS on-column for 4 hr at 4°C, followed by elution with lysis buffer. (ii) After cleavage, the eluted FUS-NLS was further purified by gel filtration chromatography with a Superdex 200 HR 10/30 column (GE Healthcare). Mutations in FUS-NLS were generated by site-directed mutagenesis and the mutant proteins were expressed and purified as described above.

To prepare the Trn1/FUS-NLS complex, purified Trn1 and FUS-NLS were mixed in a molar ratio of 1∶2 and kept on ice for 2 h. The Trn1/FUS-NLS complex was then concentrated to 5 mg/ml for crystallization.

### Crystallization, Data Collection, and Structure Determination

Hanging drops were made by mixing a solution (2 µl) containing the FUS-NLS/Trn1 complex (5 mg/ml protein in 20 mM HEPES, pH 7.3, 110 mM potassium acetate, 10 mM DTT) with an equal volume of reservoir solution containing 640 mM potassium-sodium tartrate and 20 mM HEPES buffer, pH 7.4. Crystals with a size of 200 µm × 50 µm × 10 µm were grown at 289°K within two weeks. Harvested crystals were cryoprotected with a reservoir solution supplemented with 26% (v/v) glycerol and then mounted for flash-cooling at 100°K. Diffraction data were collected at the beamline BL17U1 of Shanghai Synchrotron Radiation Facility (SSRF) (Shanghai, China) using an MX225 CCD detector. Data processing and reduction were carried out using the HKL2000 package [17]. The structure of the FUS-NLS/Trn1 complex was solved first by molecular replacement with Molrep from CCP4 suite [18] using the atomic coordinates of human Trn1 (PDB code: 2Z5J) [15] as a search model. Molecular-replacement solutions were modified and refined with alternate cycles of manual refitting and building into a 2*F*_o_ − *F*_c_ composite omit electron density map using Coot [19] and simulated annealing and maximum likelihood protocols using CNS [20], REFMAC [21], and phenix.refine [22]. The final model of the complex was checked for geometrical correctness with PROCHECK [23]. In the final model, the electron densities for residues 1–4 and 323–371 of the Trn1 and residues 495–507 of the FUS-NLS were invisible, and these 66 residues were excluded from the model. Furthermore, because of the poor electron densities for the side chains of residues K6, D8, R870 and R871 in Trn1, these 4 residues were mutated to Alanine in the model.

Cartoon and surface representations were generated using PyMOL. The electrostatic potential was calculated and displayed with PyMOL. The atomic coordinates and structural factors for the human Trn1/FUS-NLS complex have been deposited in the Protein Data Bank (PDB) database with accession code 4FQ3.

### Surface Plasmon Resonance Analysis (SPR)

All surface plasmon resonance experiments were carried out using a BIAcore 3000 biosensor (GE Healthcare) at 25°C. Trn1 was immobilized on a CM5 sensor chip by the amino coupling method to give about 8600 Resonance Unit (RU). WT and mutant FUS-NLS were prepared in the running buffer (20 mM HEPES, pH 7.3, 110 mM potassium acetate, 2 mM magnesium acetate, 2 mM EGTA, 2 mM DTT, 10% glycerol and 0.005% [v/v] Tween 20) and injected at increasing concentrations at a flow rate of 50 µl/min. The association was allowed to proceed for 60 sec, and the dissociation of the complex was monitored for 60–360 sec. Regeneration of the chip was done with 25 µl of 2 M NaCl followed by 50 µl of the running buffer.

For the WT FUS-NLS and mutants G507D, S513P, G515C, R518K, R521G, E523S and R524S, the 1∶1 Langmuir model was applied to fit the experimental results to calculate the affinity (*K*_D_) and kinetics (*k*_a_ and *k*_d_) of the FUS-NLS/Trn1 binding. For mutants K510E, R514G, H517P, R518G, R522G, P525L and Y526A, the interaction with Trn1 was weak and the *k*_d_ was too fast to be fit. Thus, the *K*_D_ values of these weak interactions were calculated by plotting the steady state equilibrium binding as a function of the concentration of the injected proteins. The correlation coefficient χ^2^ value is a statistical measure of how closely the fitted curve fits the experimental data. In general, χ^2^ values lower than about 10 signify a good fit [15].

In the particular case of the E523Y mutant, its binding to Trn1 was so tight that the dissociation of the complex was not observed under the experimental conditions (Figure S3). Thus, we were unable to obtain the affinity and kinetics constants of the interaction of Trn1 with the E523Y mutant of FUS-NLS.

### Confocal Microscopy

Confocal microscopy was used to examine the subcellular localization of WT full-length human FUS and 5 different ALS mutants (S513P, G515C, R521G, R522G and P525L). Neuroblastoma 2a (N2a) cells were seeded into 12-well plate with gelatin-coated 18-mm coverslips inside. Various GFP-FUS constructs were generated and transfected into N2a cells using Lipofectamine 2000 (Invitrogen) as previously published [24]. Alternatively, primary dorsal root ganglion (DRG) neurons were prepared and used as previously published [10]. 24 hours after transfection, cells were fixed in 4% paraformaldehyde, permeabilized by 0.1% Triton X-100. The nuclei were stained by 4′,6-diamidino-2-phenylindole (DAPI). The coverslips were mounted and images were acquired using an Olympus confocal microscope (Olympus Fluoview, Ver.1.7c).

## Results

### Extensive Interaction between FUS-NLS and Trn1 in the Trn1/FUS-NLS Complex

The structure of the binary complex consisting of human Trn1 (residues 1–890) and human FUS-NLS (residues 495–526) was determined by X-ray crystallography (PDB code: 4FQ3). The orthorhombic crystal (space group *P*2_1_2_1_2) contains one complex per asymmetric unit. The data-collection and refinement statistics are summarized in Table S1.

**Figure 2:**
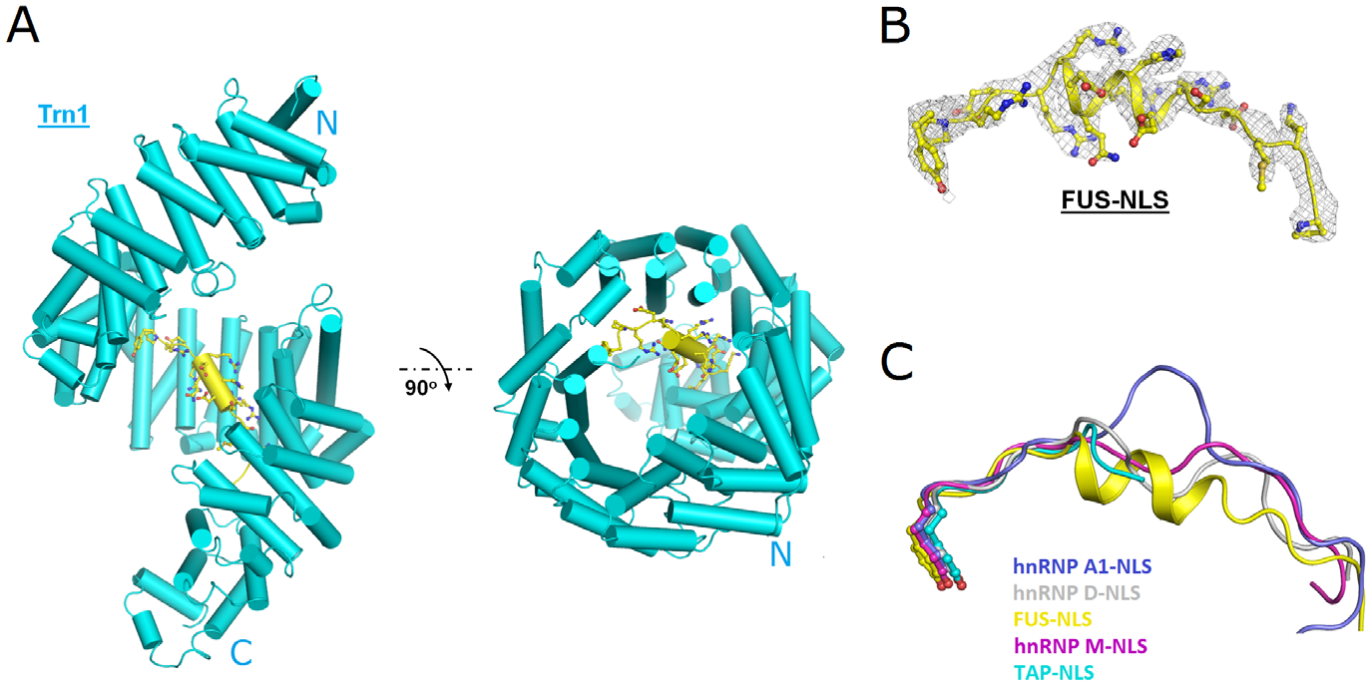
The structure of the FUS-NLS/Trn1 complex. (A) The overall structure. (B) The 2*F_o_* − *F_c_* composite omit electron density map around the FUS-NLS fragment (residues 508–526) contoured at 1.0 σ (gray mesh). The Trn1 and the FUS-NLS are shown in cyan and yellow, respectively. (C) The superimposition of residues 508–526 of FUS-NLS (yellow; PDB code: 4FQ3) with the corresponding regions from hnRNP A1-NLS (blue; PDB code: 2H4M), hnRNP D-NLS (grey; PDB code: 2Z5N), hnRNP M-NLS (magenta; PDB code: 2OT8), and TAP-NLS (cyan; PDB code: 2Z5K). The α-helix is unique in FUS-NLS whereas no specific secondary structure was found in the other structures.

Within the complex, the structure of Trn1 is highly helical and forms a perfect right-handed solenoid structure with 20 HEAT repeat domains (Figure 2A), which is similar to what have been published [13]–[15]. A C-terminal arch formed by HEAT repeats 8–18 of Trn1 is responsible for binding with FUS-NLS. When the Trn1 structure determined in this study (PDB code: 4FQ3) was superimposed onto that in the Trn1/hnRNP A1-NLS, Trn1/hnRNP D-NLS, Trn1/hnRNP M-NLS and Trn1/TAP-NLS complexes (PDB codes: 2H4M, 2Z5N, 2OT8 and 2Z5K) [13]–[15], the atomic r.m.s.d. values are 6.05 Å, 3.15 Å, 3.62 Å and 3.31 Å, respectively.

**Figure 3:**
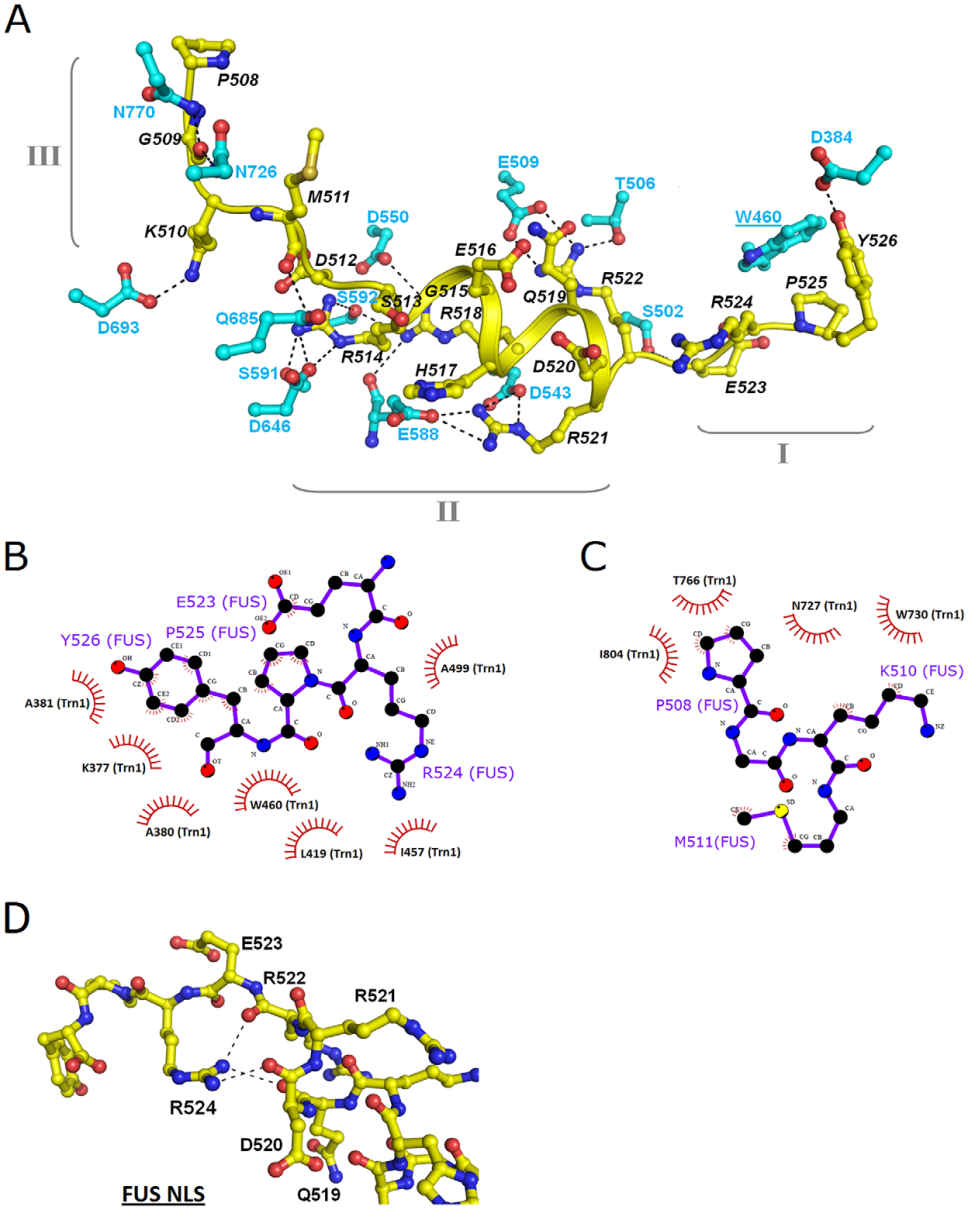
The interactions between the Trn1 and the FUS-NLS. (A) Summary of the polar/electrostatic interactions between FUS-NLS (yellow) and Trn1 (cyan). FUS-NLS is divided into region I (E523–Y526), region II (D512–R522), and region III (P508–M511). (B) Schematic illustration of the hydrophobic contacts between the region I of FUS-NLS (E523–R524–P525–Y526) and Trn1. (C) Schematic illustration of the hydrophobic contacts between the region III of FUS-NLS (P508–G509–K510–M511) and Trn1. (D). The interaction between R524 and Q519, D520 and R522 within FUS-NLS. The figure is prepared with LIGPLOT [38].

FUS-NLS forms a well-organized structure in the complex in this study (Figure 2B) as compared to other PY NLS’s with no specific secondary structure in previous studies. In particular, the α-helix (R514–R521) within FUS-NLS is not formed in other PY NLS’s (Figure 2C). These structural features facilitate the extensive interactions with Trn1. Based on the structural features and the nature of the interaction, we divide FUS-NLS into three regions: region I (E523–Y526), region II (D512–R522), and region III (P508–M511). These regions and the Trn1 residues they interact with are shown in Figure 3A.

Region I of FUS-NLS is involved in docking into the PY-motif recognition pocket in Trn1 through hydrophobic contacts. Residues E523, P525, and Y526 of FUS-NLS are in contact with K377, A381, L419, I457, W460 and A499 in Trn1 (Figure 3B, S1A and Table S2). In addition, a hydrogen bond between Y526 of the FUS-NLS and D384 of Trn1 further enhances the interaction (Figure 3A and Table S3).

Region II of FUS-NLS is unique as it forms an α-helix structure that cannot be found in other PY NLS’s (Figure 2C). The helical structure makes the side chains of R514, H517, R518, R521 and R522 face outwards, displaying a continuously positively charged patch (Figure S1B and S1D). These 5 positively charged residues of FUS-NLS are involved in polar and electrostatic interactions with acidic and polar residues (T506, E509, D543, D550, E588, S591, S592 and D646) on the surface formed by HEAT Repeats 11–14 of Trn1 (Figures 3A, S1B and Table S3).

Region III of FUS-NLS interacts with Trn1 mainly through hydrophobic interactions. Residues P508, K510 and M511 in FUS-NLS are in contact with residues N726, N727, W730, T766 and I804 in Trn1 (Figures 3C, S1C and Table S2). In addition, hydrogen bonds and ion pairs between residues G509 and K510 in FUS-NLS and residues N726, N770, and D693 in Trn1 also contribute to the interaction (Figure 3A and Table S3).

The extensive contacts between FUS-NLS and Trn1 are generally categorized as hydrophobic and polar/electrostatic interactions and summarized in Tables S2 and S3, respectively. It becomes evident that regions I and III interact with Trn1 mainly by hydrophobic forces and region II by polar/electrostatic interaction.

### Structural Characteristics of the C-terminal PY-fragment of FUS-NLS

The perfect docking of the PY-containing region I of FUS-NLS into the hydrophobic PY-motif recognition pocket of Trn1 is critical. To form the specific conformation required for docking to Trn1, the hydrophobic patch formed by the hydrophobic parts of the side chains of E524, P525 and Y526 residues is required. Figure S2A shows the superimposition of region I (E523–R524–P525–Y526, the PY motif) of FUS-NLS with the corresponding motif in hnRNP D-NLS (Y352–K353–P354–Y355; PDB code: 2Z5N), hnRNP M-NLS (F61–E62–P63–Y64; PDB code: 2OT8), hnRNP A1-NLS (S286–G287–P288–Y289; PDB code: 2H4M) and TAP-NLS (Y72–N73–P74–Y75; PDB code: 2Z5K). It is evident that the structure of this motif is highly conserved. At the sequence level, although P525 and Y526 are absolutely conserved, the other two residues vary substantially among the five PY NLS’s.

The R524 residue in FUS-NLS is unique in that it provides additional steric constraints for region I by forming hydrogen bonds with neighboring residues. As shown in Figure 3D, the hydrogen bonds between R524(N^η1^) and R522(O), R524(N^η2^) and D520(O), and R524(N^η1^) and Q519(O) assist region I to maintain a rigid conformation. In contrast, G287 in hnRNP A1 (Figure S2A) and N73 in TAP (Figure S2B) form none or 1 hydrogen bond in their structures, respectively. The combination of P525 and R524 makes the PY-motif (region I) a rigid structure that is optimized for recognition by Trn1.

### Binding Affinity of Wild-type and Mutant FUS-NLS with Trn1

To quantitatively analyze the binding affinity between FUS-NLS and Trn1, we performed surface plasmon resonance (SPR) to measure the dynamics of the interaction between FUS-NLS and Trn1. In addition to wild-type FUS-NLS (referred as WT), we also measured 12 ALS-associated mutants: G507D, K510E, S513P, R514G, G515C, H517P, R518G, R518K, R521G, R522G, R524S and P525L [7], [8]. Three additional mutations were designed based on the structural insights from this study (E523S, E523Y and Y526A) and have not been found in familial ALS patients yet.

The SPR results for WT FUS-NLS and all mutants are summarized in Table 1. The binding affinity between WT and Trn1 is strong with a dissociation constant (*K*_D_) of 1.7×10^−9^ M. All 12 ALS-associated point mutations reduced the binding affinity and the reduction varies widely in the range of 1.4- to 714-fold.

**Table 1:** Association rate, dissociation rate, and equilibrium dissociation constants of Trn1 and wild-type and mutant FUS-NLS.

Immobilized	Analyte	*k*_a_ (M^−1^s^−1^)	*k*_d_ (s^−1^)	*K*_D_ (M)	Relative affinity^a^	χ^f^
	FUS-NLS(WT)	2.3×10^6^	3.7×10^−3^	1.7×10^−9^	1	10.3
	FUS-NLS(S513P)	9.4×10^6^	2.3×10^−2^	2.4×10^−9^	0.71	5.78
	FUS-NLS(G515C)	1.8×10^6^	5.1×10^−3^	2.8×10^−9^	0.61	7.84
	FUS-NLS(G507D)	5.7×10^6^	4.4×10^−2^	7.8×10^−9^	0.22	2.04
	FUS-NLS(R524S)	5.6×10^6^	5.8×10^−2^	1.0×10^−8^	0.17	1.75
	FUS-NLS(R518K)	6.0×10^6^	7.8×10^−2^	1.3×10^−8^	0.13	2.85
	FUS-NLS(R521G)	1.4×10^5^	3.4×10^−3^	2.5×10^−8^	0.068	17.30
Trn1	FUS-NLS(R514G)			6.7×10^−8^	0.025	2.85
	FUS-NLS(H517P)			1.4×10^−7^	0.012	5.14
	FUS-NLS(R518G)			1.5×10^−7^	0.011	0.97
	FUS-NLS(K510E)			1.5×10^−7^	0.011	9.79
	FUS-NLS(R522G)			3.5×10^−7^	0.0049	0.78
	FUS-NLS(P525L)			1.2×10^−6^	0.0014	2.67
	FUS-NLS(Y526A)			8.9×10^−7^	0.0019	11.70
	FUS-NLS(E523S)	5.5×10^3^	1.1×10^−5^	2.0×10^−9^	0.85	5.50
	FUS-NLS(E523Y)	–	–	–	–	

The 12 ALS mutations are organized in the order of decreasing affinity.

a The relative affinity is defined as *K*_D_ of WT (M) divided by *K*_D_ of the FUS-NLS mutants *(*M*).*

The correlation coefficient χ^2^ value is a statistical measure of how closely the fitted curve fits the experimental data [15] (see Methods).

Among the ALS mutations, P525L reduces the affinity most significantly (more than 700× reduction) followed by R522G (∼200× reduction). In contrast, S513P or G515C only causes a slight decrease of the binding affinity (less than 2× reduction). When mutation occurs to the positive residues in the α-helix of region II, each of the single mutation (R514G, H517P, R518G, R521G and R522G) caused significant reduction (from ∼8× to 204×) in the binding affinity (Table 1).

Based on the structure of the FUS-NLS/Trn1 complex, we designed three additional mutants (Y526A, E523S and E523Y) and measured their binding affinity with Trn1. Y526A causes ∼500× reduction in the affinity, which is expected since Y526 is critical in the PY NLS. E523 is interesting since it is the most variable residue in region I (Figure S2A). The C^δ^ of E523 is involved in a hydrophobic interaction with C^α^ and C^β^ of A499 in Trn1 (Table S2). We predicted that E523Y could enhance the interaction and that E523S could reduce the affinity. In the SPR analysis, the E523Y mutant bound with Trn1 on the chip so strongly that it could not be dissociated (Figure S3), thus we could not obtain the dissociation constant. On the other hand, E523S mutation indeed caused ∼20% reduction in the *K*_D_ value.

### Impairment of Nuclear Targeting is Correlated with FUS-NLS/Trn1 Binding Affinity

**Figure 4:**
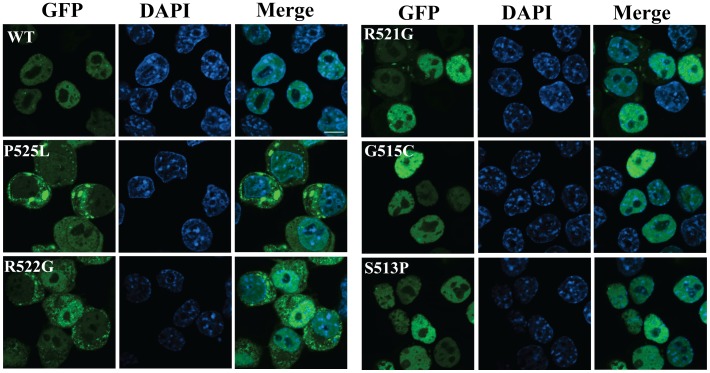
Subcellular localization of WT and mutant FUS. GFP-tagged WT full-length human FUS or different ALS mutants (S513P, G515C, R521G, R522G and P525L) were transfected into N2a cells. The cells were fixed in 4% paraformaldehyde and permeabilized by 0.1% Triton X-100 24 hours after transfection. The nuclei were stained by 4′,6-diamidino-2-phenylindole (DAPI). The coverslips were mounted and images were acquired using an Olympus confocal microscope (Olympus Fluoview, Ver.1.7c).

The regulation of subcellular localization of FUS is critical to maintain its proper function and the aberrant cytoplasmic accumulation of FUS is a prominent feature in ALS. We rationalize that ALS mutations with different reduction levels in the FUS-NLS/Trn1 binding affinity will have different effects on the subcellular localization of FUS *in vivo*. We tested this hypothesis by examining the subcellular localization of WT, P525L, R522G, R521G, S513P and G515C full-length FUS in N2A cells as well as primary neurons. As shown in Figure 4, WT FUS is predominantly inside the nucleus. For the P525L and R522G mutations that disrupt the FUS-NLS/Trn1 binding most significantly, the mutant FUS is predominantly outside the nucleus and forms cytoplasmic inclusions. For the S513P and G515C mutations that minimally disrupt the FUS-NLS/Trn1 binding, the mutant FUS is still predominantly inside the nucleus. For R521G that causes approximately 15× reduction in the FUS-NLS/Trn1 binding, the mutant protein is localized in both the nucleus and cytoplasm. Similar results were obtained in the primary dorsal root ganglia (DRG) neurons (Supplementary Figure S4). The results support that the disruption of nuclear targeting is closely correlated with the fold of reduction in the FUS-NLS/Trn1 binding.

### Correlation between FUS-NLS/Trn1 Binding Affinity and ALS Disease Duration

**Figure 5:**
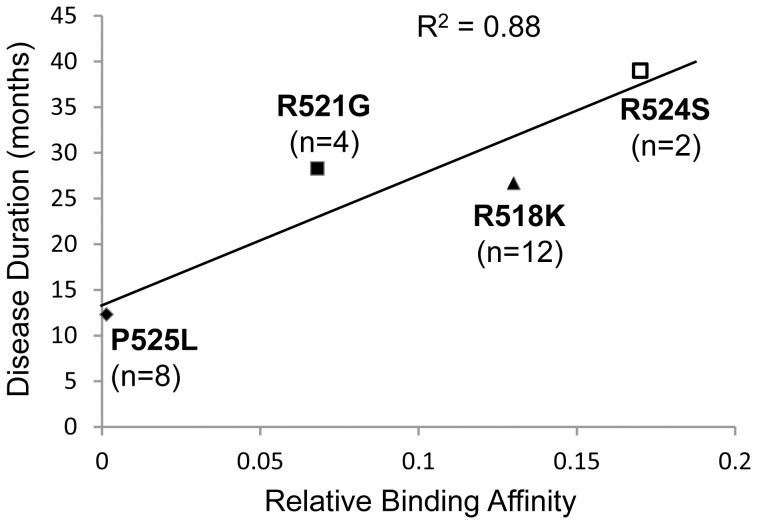
Correlation between the disease duration of familial ALS patients carrying R518K, R521G, R524S and P525L mutations and the relative binding affinity of the mutant proteins to Trn1. The coefficient of determination R^2^ is 0.88, suggesting strong correlation. The number of patients for the R518K, R521G, R524S and P525L mutations are 12, 4, 2, and 8, respectively.

We further asked whether the FUS-NLS/Trn1 binding affinity can possibly correlate with the ALS disease manifestation in human patients. The durations of the disease from onset in the familial ALS patients carrying different FUS mutations were compiled from published studies [7], [25]–[28] and plotted against the relative affinity of the corresponding mutant from Table 1. As shown in Figure 5, the disease duration correlates very well with the relative affinity and the coefficient of determination R^2^ is 0.88. It is suggested that the greater disruption in FUS-NLS/Trn1 interaction, the greater level of FUS mis-localization, and the more rapid disease progression in the patient carrying the particular mutation.

## Discussion

This study describes a structural, biochemical and cell biological characterization of FUS-NLS that we and others previously identified [10]–[12]. We first determined the crystal structure of the FUS-NLS/Trn1 complex, and then measured the binding affinity of WT and mutant FUS-NLS to Trn1. We also examined the subcellular localization of WT and mutant full-length FUS and showed a strong correlation between the reduced binding affinity and increased FUS mis-localization. We last discuss the implication of this study in understanding ALS etiology and future therapeutic development.

As discussed earlier, cytoplasmic accumulation and pathological inclusions of FUS are prominent features in ALS. The RNA and DNA targets of FUS have been reported recently [29], [30] and the nucleic acid binding also requires the nuclear localization of FUS. The data suggest that it is critical to understand the detailed nuclear targeting mechanism.

Although there are some similarities, the interaction of FUS-NLS with Trn1 differs in many ways from that of Trn1 with other PY NLS’s in previous studies [13]–[15]. FUS-NLS forms extensive contact with Trn1 and we categorized the FUS-NLS into three regions based on the nature of the interaction: region I (PY-fragment), region II (the helical region) and region III (the hydrophobic motif). Region I and III mainly interact with Trn1 by hydrophobic interaction (Figures 3B, 3C, S1A, S1C and Table S2) whereas region II mainly by polar/electrostatic interaction (Figure 3A and Table S3). These extensive interactions account for the underlying mechanism for the high affinity binding of FUS-NLS to Trn1 (*K*_D_ = 1.7 nM).

The most remarkable distinction between FUS-NLS and other PY NLS’s is the α-helix in region II (Figure 2B and 2C). This α-helix exposes all positively charged residues (R514, H517, R518, R521 and R522) in the region, which allows them to form electrostatic contacts with the negatively charged surface of Trn1 (Figure S1B and S1D). Consequently, the ALS-associated mutations in this region (R514G, H517P, R518G, R521G and R522G) caused significantly decreased binding affinity with Trn1 (∼8× to 204× reduction).

Within the short region I (the PY fragment, E523-Y526), P525 and Y526 are the most important residues. The ALS mutation P525L and the Y526A mutation we generated both dramatically decreased the binding affinity. In addition, FUS-NLS utilizes E523 and R524 to maintain the specific conformation of the PY fragment that will allow the optimized interaction/recognition. E523 is unique in that its C^δ^ forms hydrophobic interaction with C^α^ and C^β^ of A499 in Trn1 (Table S2). There is no ALS-associated mutation reported on E523 yet. As described earlier, R524 forms three pairs of hydrogen bonds with R522, D520 and Q519 (Figure 3D), which significantly enhances the rigidity of the PY fragment to facilitate the interaction with Trn1. Such features are not observed in other PY NLS’s. Consequently, the ALS mutation R524S causes an approximately 6× reduction in the binding affinity (Table 1) although R524 does not directly interact with Trn1. This suggests that R524 and the hydrogen bonds it forms are important. As a comparison, S513 and G515 are also not directly involved in the FUS-NLS/Trn1 interaction and S513P and G515C only cause less than 2-fold reduction in the binding affinity (Table 1).

It is noted that the FUS-NLS is highly conserved in many organisms (Figure 1C). It is logical that the NLS is critical to the proper subcellular localization and function of FUS. Our subcellular localization results from WT full-length FUS and five different ALS-associated mutants clearly demonstrated the correlation between binding affinity and nuclear targeting efficiency (Figure 4). When a mutation causes greater reduction in the FUS-NLS/Trn1 binding affinity, it will induce greater cytoplasmic accumulation of FUS. Different ALS-associated mutants showing varied FUS-NLS/Trn1 binding affinities caused different extents of cytoplasmic accumulation.

This logically leads to a critical question: is the binding affinity possibly correlated with the ALS disease manifestation in human patients? We have shown that the disease duration correlates very well with the relative affinity (Figure 5), suggesting that the greater disruption in the FUS-NLS/Trn1 interaction, the greater level of FUS mis-localization, and the more rapid disease progression in the patient carrying the particular mutation. In another study that reported S513P and H517P mutations [31], the onset in patients with the S513P mutation (the mutation that causes a minimal disruption in FUS-NLS/Trn1 binding) was around 60 years old as compared to 30 years in patients with the H517P mutation (reduced the binding affinity 83×). In addition, the case with the S513P mutation progressed slowly, but specific disease duration data were not reported [31]. These clinical observations are consistent with our results that S513P only minimally decreased the FUS-NLS/Trn1 binding affinity (∼30% reduction). Moreover, in the extreme cases of the FUS truncation mutant R495X that lacks the NLS, studies showed juvenile onset and rapid progression in these patients [26], [28]. The results support the critical importance of the NLS in maintaining normal FUS function under physiological conditions as well as the severe consequence of disrupting the NLS in ALS under pathological conditions.

It is noted that the clinical data are limited (a total of 26 patients carrying four different FUS mutations were plotted in Figure 5) and scattered in the literature and that more clinical data are needed to examine whether the correlation applies to other mutations. In addition to the intrinsic properties of FUS, it is conceivable that other factors could have significant impact on the FUS localization, downstream pathways and ultimate clinical manifestation in patients. Potential factors include environment (lifestyle, stresses) and ageing. It is possible that ageing related changes could augment the mislocalization of mutant FUS, even the mutations that have minor reduction in their binding affinity to Trn1 (e.g. S513P and G515C). It is also possible that the stress granules induced by mutant FUS [10]–[12] could exacerbate cytoplasmic accumulation of mutant FUS. These remain to be determined in future studies.

A related question is whether the relative affinity of mutant FUS is correlated with the age of disease onset/diagnosis. Despite the pair-wise comparison discussed above, systematic analysis of patients carrying S513P, H517P R518K, R521G, R524S and P525L mutations showed no apparent correlation between the disease onset age and the relative affinity of mutant FUS (R^2^ = 0.40, data not shown). It is known that ALS is a non-cell autonomous disease and the disease onset and progression are influenced by different cell types in central nerve system in the mouse models of mutant SOD1 mediated ALS [32]–[35]. It remains to be elucidated what factors determine the disease onset and progression in FUS mediated familial ALS.

As for future therapeutic development, the implication of this study is to provide a detailed structural basis for designing potential small molecules that can modulate the FUS-NLS/Trn1 interaction so that the disruption of the ALS mutation can be minimized. A similar strategy has been used to design an inhibitor of nuclear import [14], thus it is conceivable that our structural data can benefit the design of compounds that can enhance nuclear import. Such small molecules that can minimize the disruptive effect of the ALS mutations could be tested first in model organisms such as *Drosophila* [36], potentially providing a new avenue for ALS treatment.

In the final days of finishing this manuscript, a FUS-NLS/Trn1 complex structure was published online [37]. The unique α-helical structure in FUS-NLS is also noted in that study. The binding affinity between wild-type FUS-NLS and Trn1 was consistent in the nM range although different techniques were used in the two studies. We measured the binding affinities for more ALS mutants and found that some mutations decreased the affinity more drastically. For instance, the interaction between P525L mutant FUS-NLS and Trn1 was found to decrease more than 700 fold in our study whereas the decrease was only 9 fold in the other study. This could potentially be due to the fact that different techniques were used; we used SPR and the other study used isothermal titration calorimetry (ITC). The correlation between the decreased affinity and the subcellular localization (Figure 4) as well as the ALS disease progression (Figure 5) is discussed in depth in our study.

## Supporting Information

Figure S1 **The surface electrostatic potential of Trn1 at the binding site interacting with region I (A), region II (B) and region III (C) of FUS-NLS.** The surface interacting with region I and region III are largely neutral whereas the surface interacting with region II is highly negatively charged. FUS-NLS was shown in yellow. (D) The surface properties of the FUS-NLS. The positively charged surface in region II interacts with the corresponding negatively charged surface in Trn1. The potential displayed represents a range from −15 (red) to +15 (blue) *k*_B_ *T*.
(PDF)

Figure S2 **Structural properties of the PY fragments of the FUS-NLS (region I) and the other PY-NLS’s.** (A) Structural alignment of the PY fragments of the FUS-NLS (yellow; PDB code 4FQ3), hnRNP A1-NLS (blue; PDB code 2H4M), hnRNP D-NLS (grey; PDB code 2Z5N), hnRNP M-NLS (magenta; PDB code 2OT8), and TAP-NLS (cyan; PDB code 2Z5K). (B) N73 and R71 of the TAP-NLS form one intramolecular hydrogen bond.
(PDF)

Figure S3 **SPR analysis of WT and E523Y mutant NLS binding with Trn1.** The E523Y mutant bound to Trn1 so tightly that the dissociation of the complex was not observed under the experimental conditions.
(PDF)

Figure S4 **Subcellular localization of WT and mutant FUS in primary mouse dorsal root ganglion (DRG) neurons.** GFP-tagged WT full-length human FUS or different ALS mutants (G515C, R522G and P525L) were transfected into DRG neurons. Cells were fixed and permeabilized 48 hours after transfection. The nuclei were stained by 4′,6-diamidino-2-phenylindole (DAPI). The coverslips were mounted and images were acquired using an Olympus confocal microscope.
(PDF)

Table S1 **Summary of data-collection and refinement statistics.**
(PDF)

Table S2 **Summary of hydrophobic contacts between Trn1 and FUS-NLS.**
(PDF)

Table S3 **Summary of polar/electrostatic interactions between Trn1 and FUS-NLS.**
(PDF)
