# Supplementary figures and images for: FUS-NLS/Transportin 1 Complex Structure Provides Insights into the Nuclear Targeting Mechanism of FUS and the Implications in ALS
Source: PLoS One. 2012 Oct 8;7(10):e47056. doi: 10.1371/journal.pone.0047056 (PMC3466232; doi:10.1371/journal.pone.0047056)

A

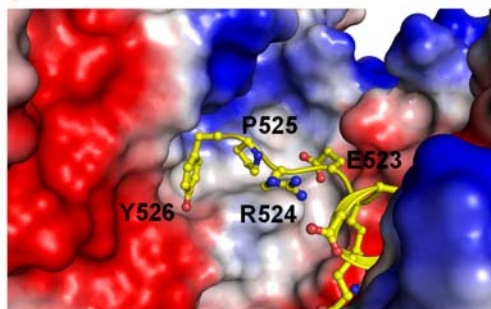

B

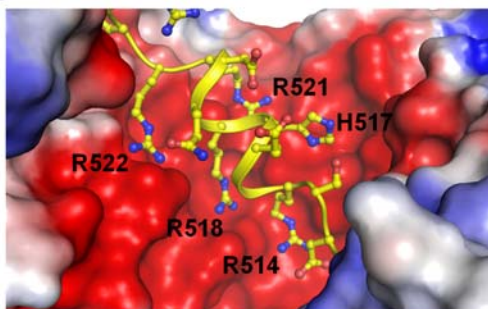

C

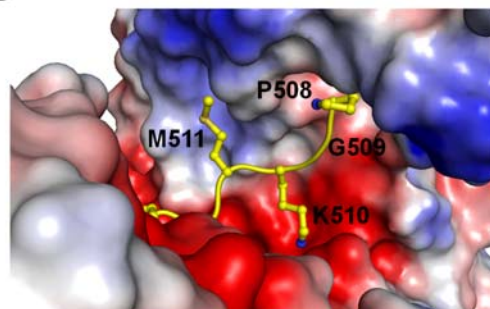

D

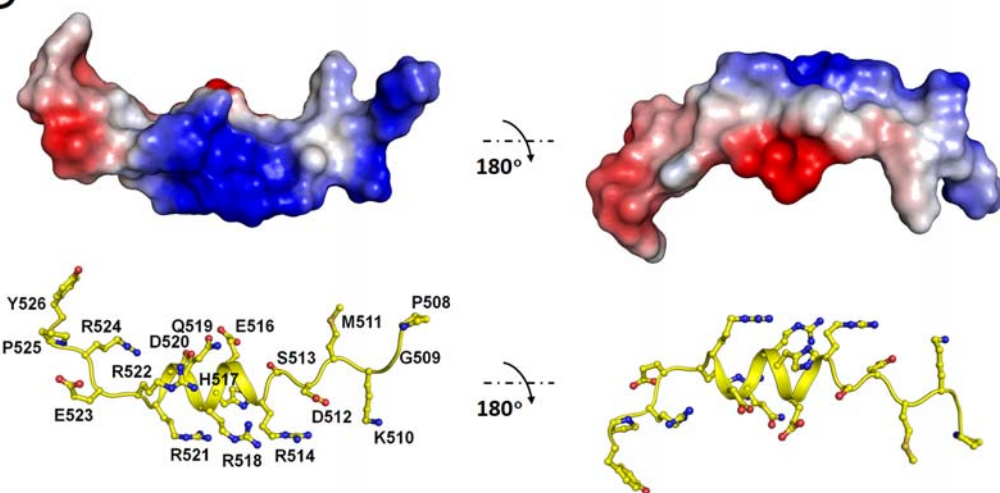

Supplement: Figure S1 — The surface electrostatic potential of Trn1 at the binding site interacting with region I (A), region II (B) and region III (C) of FUS-NLS. The surface interacting with region I and region III are largely neutral whereas the surface interacting with region II is highly negatively charged. FUS-NLS was shown in yellow. (D) The surface properties of the FUS-NLS. The positively charged surface in region II interacts with the corresponding negatively charged surface in Trn1. The potential displayed represents a range from −15 (red) to +15 (blue) k B T. (PDF) [file pone.0047056.s001.pdf]

A

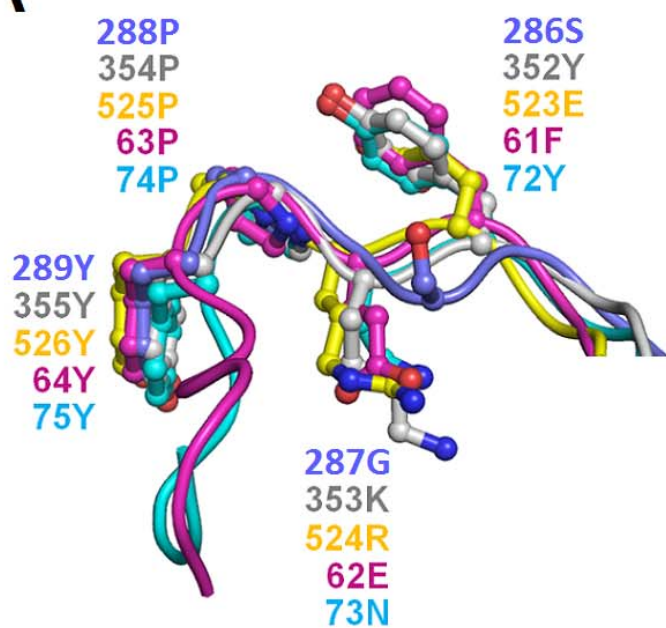

B

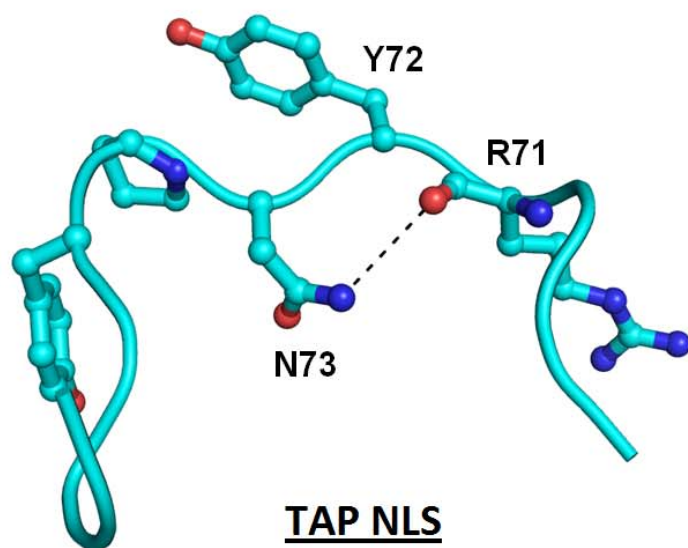

Supplement: Figure S2 — Structural properties of the PY fragments of the FUS-NLS (region I) and the other PY-NLS’s. (A) Structural alignment of the PY fragments of the FUS-NLS (yellow; PDB code 4FQ3), hnRNP A1-NLS (blue; PDB code 2H4M), hnRNP D-NLS (grey; PDB code 2Z5N), hnRNP M-NLS (magenta; PDB code 2OT8), and TAP-NLS (cyan; PDB code 2Z5K). (B) N73 and R71 of the TAP-NLS form one intramolecular hydrogen bond. (PDF) [file pone.0047056.s002.pdf]

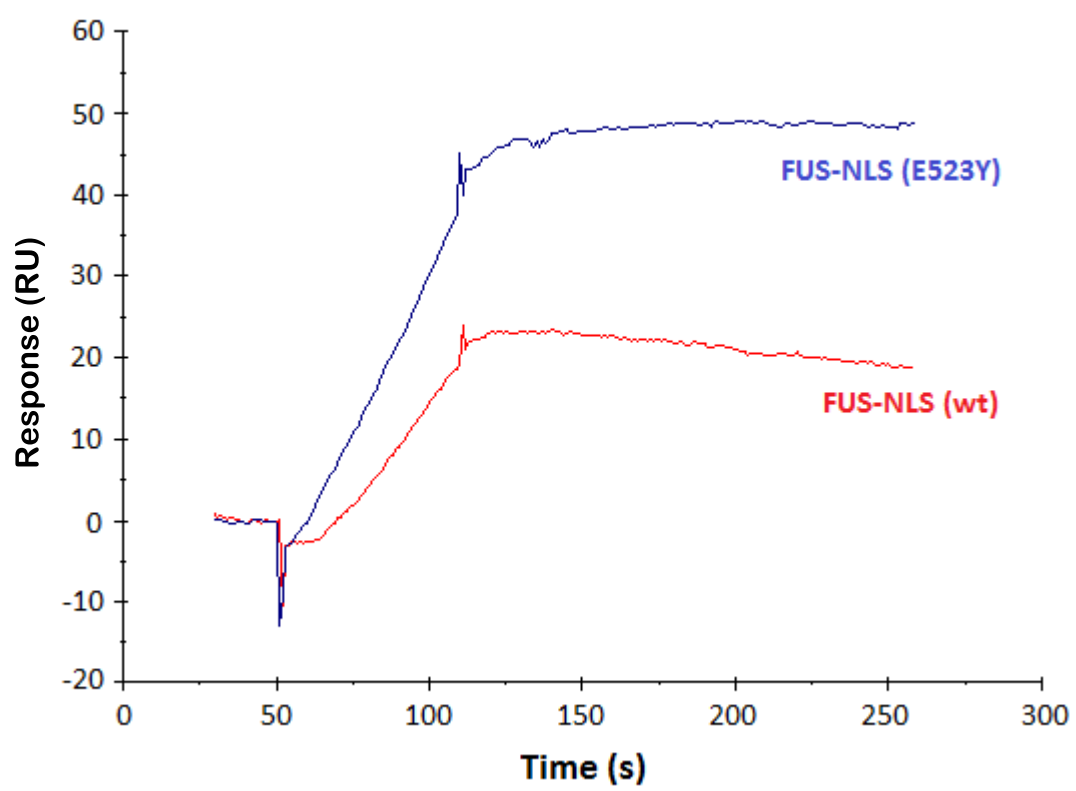

Supplement: Figure S3 — SPR analysis of WT and E523Y mutant NLS binding with Trn1. The E523Y mutant bound to Trn1 so tightly that the dissociation of the complex was not observed under the experimental conditions. (PDF) [file pone.0047056.s003.pdf]

Figure S4

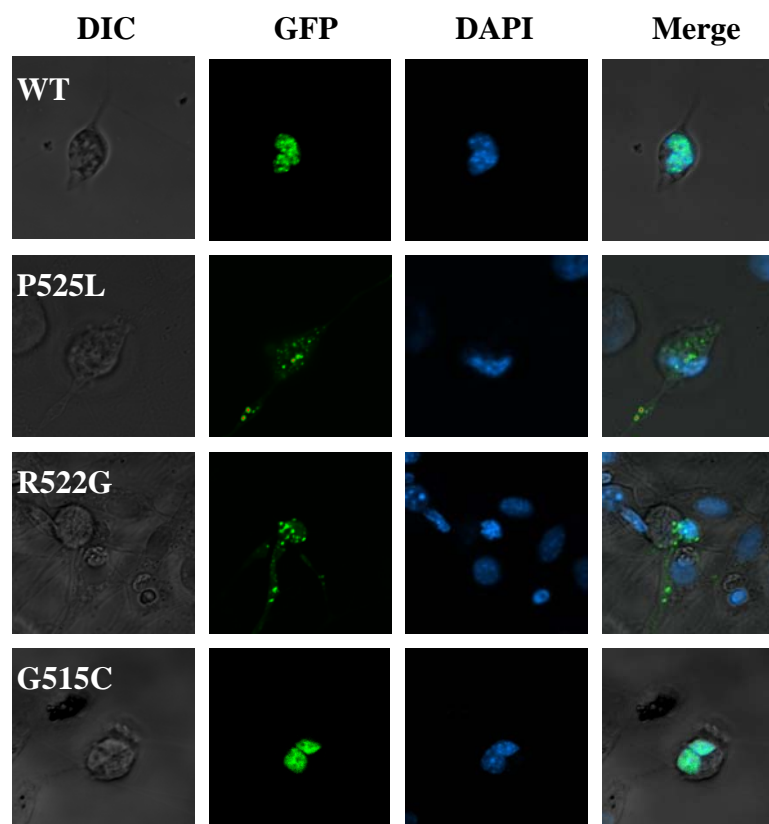

Supplement: Figure S4 — Subcellular localization of WT and mutant FUS in primary mouse dorsal root ganglion (DRG) neurons. GFP-tagged WT full-length human FUS or different ALS mutants (G515C, R522G and P525L) were transfected into DRG neurons. Cells were fixed and permeabilized 48 hours after transfection. The nuclei were stained by 4′,6-diamidino-2-phenylindole (DAPI). The coverslips were mounted and images were acquired using an Olympus confocal microscope. (PDF) [file pone.0047056.s004.pdf]
