# Supplementary material for: FUS-NLS/Transportin 1 Complex Structure Provides Insights into the Nuclear Targeting Mechanism of FUS and the Implications in ALS
Source: PLoS One. 2012 Oct 8;7(10):e47056. doi: 10.1371/journal.pone.0047056 (PMC3466232; doi:10.1371/journal.pone.0047056)
Supplement: Table S1 — Summary of data-collection and refinement statistics. (PDF) [file pone.0047056.s005.pdf]

Supplementary Table S1. Summary of data-collection and refinement statistics

**Data collection**	
Space group	$P2_12_12$
Unit-cell parameters (Å)	*a* = 128.8, *b* = 158.3, *c* = 68.4
Resolution range (Å)	50.0-3.0 (3.11-3.0)
Number of total reflections	142005 (14055)
Number of unique reflections	28798 (2811)
Data completeness (%)	99.4 (99.6)
$<I>/<\sigma(I)>$	14.7 (3.7)
R_{merge} (%) [a]	10.6 (42.7)
**Refinement**	
Resolution range used for refinement (Å)	30.0-3.0
Number of reflections used	28525
R_{cryst} / R_{free} (%) [b]	19.7 / 25.1
r.m.s.d. bond length (Å)	0.006
r.m.s.d. bond angles (°)	1.06
Number of atoms modeled	6806
Mean *B* factor for protein atoms (Å^2)	19.5
**Ramachandran plot statistics**	
Residues in most favored region (%)	96.1
Residues in additional allowed region (%)	3.9
Residues in disallowed region (%)	0

[a] $R_{merge} = \sum_{hkl}\sum_i |I_i(hkl) - <I(hkl)>| / \sum_{hkl}\sum_i I_i(hkl)$, where $<I(hkl)>$ is the main value of $I(hkl)$.

[b] $R_{cryst} = \sum||F_{obs}| - |F_{calc}||/\sum|F_{obs}|$, where F_{obs} and F_{calc} are observed and calculated structure factors.

The free *R* factor was calculated using 5% of reflections omitted from the refinement.

Numbers in parentheses represent the value for the highest resolution shell.
