# Supplementary material for: FUS-NLS/Transportin 1 Complex Structure Provides Insights into the Nuclear Targeting Mechanism of FUS and the Implications in ALS
Source: PLoS One. 2012 Oct 8;7(10):e47056. doi: 10.1371/journal.pone.0047056 (PMC3466232; doi:10.1371/journal.pone.0047056)
Supplement: Table S2 — Summary of hydrophobic contacts between Trn1 and FUS-NLS. (PDF) [file pone.0047056.s006.pdf]

Supplementary Table S2. Summary of hydrophobic contacts between Trn1 and FUS-NLS

	**FUS-NLS**		**Trn1**
Region I	E523(C^{δ})	↔	A499(C^{α} and C^{β})
	P525(C^{δ})	↔	W460($C^{\zeta 2}$, $C^{\delta 1}$, $C^{\delta 2}$, $C^{\varepsilon 2}$, and $C^{\varepsilon 3}$)
	P525(C^{γ})	↔	W460($C^{\delta 2}$, $C^{\varepsilon 3}$, and C^{γ})
	P525(C^{γ})	↔	I457(C^{δ})
	P525(C^{γ} and C^{β})	↔	L419($C^{\delta 1}$)
	Y526(C^{ζ} and $C^{\varepsilon 2}$)	↔	W460($C^{\eta 2}$)
	Y526($C^{\varepsilon 2}$)	↔	L419($C^{\delta 1}$)
	Y526($C^{\delta 1}$)	↔	A381(C^{α})
	Y526($C^{\varepsilon 2}$, C^{γ}, and $C^{\delta 2}$)	↔	A381(C^{β})
	Y526(C^{β})	↔	K377(C^{α}, C^{β}, and C^{γ})
Region III	P508(C^{δ} and C^{γ})	↔	I804(C^{δ})
	K510(C^{δ})	↔	W730($C^{\eta 2}$)
	K510(C^{β})	↔	W730($C^{\varepsilon 3}$)
	K510(C^{β})	↔	N727(C^{γ})
	M511(C^{ε})	↔	T766($C^{\gamma 2}$ and C^{β})
	M511(C^{γ})	↔	N726(C^{γ})
