# Supplementary material for: FUS-NLS/Transportin 1 Complex Structure Provides Insights into the Nuclear Targeting Mechanism of FUS and the Implications in ALS
Source: PLoS One. 2012 Oct 8;7(10):e47056. doi: 10.1371/journal.pone.0047056 (PMC3466232; doi:10.1371/journal.pone.0047056)
Supplement: Table S3 — Summary of polar/electrostatic interactions between Trn1 and FUS-NLS. (PDF) [file pone.0047056.s007.pdf]

Supplementary Table S3. Summary of polar/electrostatic interactions between Trn1 and FUS-NLS

	FUS-NLS		Trn1
**Region I**	Y526(O^{η})	↔	D384($O^{\delta2}$)
**Region II**	R514($N^{\eta2}$)	↔	S591(O^{γ})
	R514($N^{\eta2}$)	↔	D646($O^{\delta2}$)
	R514(N^{ε})	↔	D646($O^{\delta2}$)
	H517($N^{\delta1}$)	↔	E588($O^{\varepsilon2}$)
	R518($N^{\eta2}$)	↔	E588(O)
	R518($N^{\eta2}$)	↔	S592(O^{γ})
	R518($N^{\eta1}$)	↔	D550($O^{\delta1}$)
	R521($N^{\eta2}$)	↔	E588($O^{\varepsilon1}$)
	R521($N^{\eta2}$)	↔	D543($O^{\delta2}$)
	R521(N^{ε})	↔	D543($O^{\delta2}$)
	R522($N^{\eta1}$)	↔	T506($O^{\gamma1}$)
	R522($N^{\eta1}$)	↔	E509($O^{\varepsilon1}$)
	R522($N^{\eta2}$)	↔	E509($O^{\varepsilon2}$)
**Region III**	G509(O)	↔	N726($N^{\delta2}$)
	G509(O)	↔	N770($N^{\delta2}$)
	K510(N^{ζ})	↔	D693($O^{\delta2}$)
